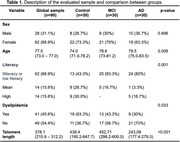# Brazilian older adults with Alzheimer’s Disease Exhibit Telomere Shortening

**DOI:** 10.1002/alz.092662

**Published:** 2025-01-03

**Authors:** Guilherme Fonseca Graciano, Thayana Oliveira Soares, Marco Túlio Cintra, Olívio Brito Malheiro, Luciano Pimenta Júnior, Samuel Oliveira Milagre, Letícia Jabur Ribeiro, Leonardo Ryuiti Kimoto, Aline Siqueira de Souza, João Henrique Fonseca, Nuno de Andrade Rezende Lima, Camila Dias Rocha, Bernardo M Viana, Ann Kristine Jansen, Rodrigo Ribeiro dos Santos, Maria Aparecida Camargos Bicalho

**Affiliations:** ^1^ Federal University of Minas Gerais, Belo Horizonte, Minas Gerais Brazil

## Abstract

**Background:**

The Brazilian population has been experiencing an increase in the number of older adults, with a simultaneous rise in the incidence of Mild Cognitive Impairment (MCI) and Alzheimer’s Disease (AD). Telomeres are structures located at the ends of chromosomes that maintain the structural integrity of the chromosome. There is a shortage of studies correlating telomeres and cognition. This study aimed to assess the relationship between telomere length and different cognitive profiles.

**Method:**

A cross‐sectional study was conducted at the Outpatient Reference Center for Older Adults at the University Hospital of the Universidade Federal de Minas Gerais (UFMG), Brazil. The sample consisted of older adults without cognitive impairment (control group), those with MCI, and those with AD`s dementia (ADD). Participants underwent geriatric and neuropsychological evaluation for clinical and cognitive assessment. DNA analysis from peripheral blood samples was performed using a saline method. Telomere relative length was analyzed using RT‐PCR. Multivariate linear regression analysis was conducted to examine the association between telomere relative length and the sample.

**Result:**

The sample comprised 90 participants, with 30 individuals in each group (control, MCI, and AD). The majority of the sample comprised women (68.9%), with an average education of 4.56 ± 3.70 years. The percentage of women was higher in all groups. The ADD group had a higher average age compared to the others, with a mean age of 79.5 (75.0‐83.5), p = 0.0060. Education was lower in the MCI group compared to the control group (25, p = 0.001). A shorter telomere length was observed in the ADD group (225.51), followed by the control group (428.27) and the MCI group (448.05) (p <0.001). The variable dyslipidemia was significantly associated with telomere length (p = 0.034). Other variables did not differ significantly.

**Conclusion:**

It was observed that the relative telomere length was associated with the ADD, with a smaller telomere relative length in this group compared to the others, and an association with dyslipidemia.